# The Pre-Diabetes Interventions and Continued Tracking to Ease-out Diabetes (Pre-DICTED) program: study protocol for a randomized controlled trial

**DOI:** 10.1186/s13063-021-05500-5

**Published:** 2021-08-06

**Authors:** Kar-Fu Yeung, Mihir Gandhi, Amanda Yun Rui Lam, Selly Julianty, Alvin Yeow Meng Chia, Gilbert Choon Seng Tan, Su-Yen Goh, Emily Tse Lin Ho, Angela Fang Yung Koh, Gavin Siew Wei Tan, Eugene Jin Wen Shum, Eric A. Finkelstein, Tazeen H. Jafar, Yee Leong Teoh, Rob M. van Dam, Clare Whitton, Julian Thumboo, Yong Mong Bee

**Affiliations:** 1 Centre for Quantitative Medicine, Duke-NUS Medical School, Singapore, Singapore; 2 Biostatistics, Singapore Clinical Research Institute, Singapore, Singapore; 3 The Center for Child Health Research, Tampere University, Tampere, Finland; 4 Department of Endocrinology, Singapore General Hospital, Singapore, Singapore; 5 SingHealth Duke-NUS Diabetes Centre, Singapore, Singapore; 6 Regional Health System Office, SingHealth, Singapore, Singapore; 7 SingHealth Polyclinics, Singapore, Singapore; 8 Department of Internal Medicine, Sengkang General Hospital, Singapore, Singapore; 9 Surgical Retinal Department, Singapore National Eye Centre, Singapore, Singapore; 10 Health Services and Systems Research, Duke-NUS Medical School, Singapore, Singapore; 11 Saw Swee Hock School of Public Health, National University of Singapore, Singapore, Singapore; 12 Department of Rheumatology and Immunology, Singapore General Hospital, Singapore, Singapore

**Keywords:** Diabetes, Pre-diabetes, Prevention

## Abstract

**Background:**

Community-based diabetes prevention programs varied widely in effectiveness, and the intervention strategy consisting of lifestyle interventions, stepwise addition of metformin, and financial incentives has not been studied in real-world clinical practice settings. The Pre-Diabetes Interventions and Continued Tracking to Ease-out Diabetes (Pre-DICTED) trial is a pragmatic trial that aims to compare the effectiveness of a community-based stepwise diabetes prevention program with added financial incentives (intervention) versus the standard of care (control) in reducing the risk of type 2 diabetes over 3 years among overweight or obese individuals with pre-diabetes.

**Methods:**

This is an open-label, 1:1 randomized controlled trial which aims to recruit 846 adult individuals with isolated impaired fasting glucose (IFG), isolated impaired glucose tolerance (IGT), or both IFG and IGT from Singapore. Intervention arm participants attend 12 group-based sessions (2 nutrition workshops, 9 exercise sessions, and a goal-setting workshop) delivered at community sites (weeks 1 to 6), receive weekly physical activity and nutrition recommendations delivered by printed worksheets (weeks 7 to 12), and receive monthly health tips delivered by text messages (months 4 to 36). From month 6 onwards, intervention arm participants who remain at the highest risk of conversion to diabetes are prescribed metformin. Intervention arm participants are also eligible for a payment/rewards program with incentives tied to attendance at the group sessions and achievement of the weight loss target (5% of baseline weight). All participants are assessed at baseline, month 3, month 6, and every 6 months subsequently till month 36. The primary endpoint is the proportion of participants with diabetes at 3 years. Secondary endpoints include the mean change from baseline at 3 years in fasting plasma glucose, 2-hour plasma glucose, HbA1c, body weight, body mass index, physical activity, and dietary intake.

**Discussion:**

The Pre-DICTED trial will provide evidence of the effectiveness and feasibility of a community-based stepwise diabetes prevention program with added financial incentives for individuals with pre-diabetes in Singapore. The study will provide data for a future cost-effectiveness analysis, which will be used to inform policymakers of the value of a nationwide implementation of the diabetes prevention program.

**Trial registration:**

ClinicalTrials.govNCT03503942. Retrospectively registered on April 20, 2018.

**Protocol version:** 5.0 Date: 1 March 2019

**Supplementary Information:**

The online version contains supplementary material available at 10.1186/s13063-021-05500-5.

## Background

Randomized controlled trials (RCTs) have shown the effectiveness of lifestyle interventions or metformin in reducing diabetes conversion in individuals with impaired glucose tolerance (IGT) [1–3]. The Finnish Diabetes Prevention Study [1] and the US Diabetes Prevention Program (DPP) [2] both showed a relative risk reduction of 58% for lifestyle interventions, while the Da Qing IGT and Diabetes Study in China study showed a relative risk reduction of 42% [3]. The lifestyle interventions from these studies emphasized physical activity and dietary modification and included individual counseling to help participants reduce total dietary intake and improve diet quality. While the results were promising, it remains a challenge to translate these findings into real-world settings. Strategies involving multiple one-to-one patient contacts are difficult to implement and sustain on a large scale in routine healthcare settings, given competing healthcare needs and resource limitations.

Since the publication of the landmark diabetes prevention clinical trials between 1996 and 2001, a series of community-based diabetes prevention programs have been conducted [4–7]. Although these community-based programs were generally effective in preventing or delaying diabetes onset, they varied widely in effectiveness, and none matches those seen in the landmark trials [4]. One study tested the effectiveness of expert guidelines on diabetes prevention, which is lifestyle intervention with addition of metformin, when required [7]. The Diabetes Community Lifestyle Improvement Program (D-CLIP) is a randomized controlled trial in Asian Indian adults with isolated IGT, isolated impaired fasting glucose (IFG), or both IFG and IGT. The study showed that group-based lifestyle intervention plus stepwise addition of metformin led to a 32% relative risk reduction during 3 years of follow-up, compared with the standard of care [7]. A stepwise approach has the following advantages: it focuses the use of metformin to those who are likely to benefit most, i.e. those who have a suboptimal response to the lifestyle intervention and continue to show a high risk of developing diabetes. In addition, it minimizes medicalizing a pre-disease state by giving participants sufficient time to revert to normal glucose tolerance state based on lifestyle intervention alone, before introducing metformin.

Notably, with fewer resources, none of the community-based diabetes prevention programs came close to replicating the weight loss achieved in the landmark trials [8]. The steady weight regain during the study period is also a cause for concern. Weight loss appears to be the key, if not critical, intermediate outcome to reduce diabetes risk [9]. Several studies reported that weight loss is associated with the number and frequency of group sessions attended [10, 11]. In this regard, financial incentives for healthy behaviors may have a role in improving adherence and/or outcomes for participants enrolled in preventive programs [12–15]. Specifically, receipt of financial incentives was associated with higher odds of attending 9 or more DPP classes among Medicaid participants with pre-diabetes [16]. Moreover, financial incentives have been shown to improve weight loss and weight loss maintenance when combined with an evidence-based weight loss program [17–19]. The form of financial incentives may also influence the weight loss outcome. Providing a reward in the form of a lottery with a small chance of a larger payout may be more effective than fixed cash rewards, as participants tend to overestimate their chances of winning [20] and the overestimated return may serve as a stronger motivator for participants to achieve the weight loss target.

The Pre-Diabetes Interventions and Continued Tracking to Ease-out Diabetes (Pre-DICTED) program is a pragmatic randomized controlled trial with the primary objective of comparing the diabetes conversion rate at 3 years in overweight or obese men and women (body mass index (BMI) ≥ 23.0 kg/m^2^) with an elevated risk of diabetes (defined as IFG and/or IGT) between the intervention group, which receives the community-based stepwise diabetes prevention program with added financial incentives, and the control group, which receives the current standard of care. Recruitment commenced on 15 November 2017.

## Methods/Design

### Aims of study

The primary objective of this study is to evaluate the effectiveness of a community-based diabetes prevention program in reducing the diabetes conversion rate at 3 years in overweight or obese men and women (BMI ≥ 23.0 kg/m^2^) with an elevated risk of diabetes (defined as IFG and/or IGT). Secondary effectiveness objectives include the comparison of fasting plasma glucose (FPG), 2-h plasma glucose, HbA1c, weight, waist circumference, BMI, physical activity level, health status, work impairment, and diet.

### Study design

**Fig. 1:**
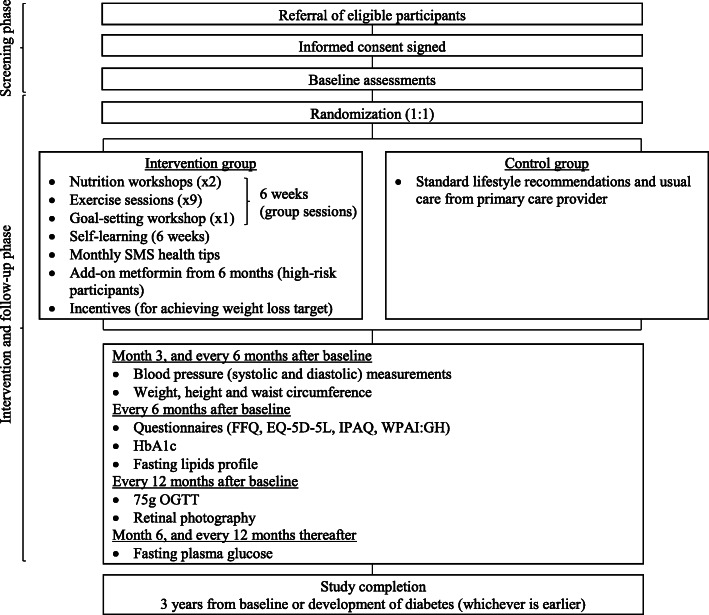
Outline of trial design

Pre-DICTED is a randomized open-label, parallel arms, controlled trial that compares a diabetes prevention program consisting of lifestyle interventions with stepwise addition of metformin and added financial incentives (intervention) versus the current standard of care (control) in overweight or obese participants with isolated IFG (FPG 6.1–6.9 mmol/L), isolated IGT (2-h plasma glucose in 75 g oral glucose tolerance test (OGTT) 7.8–11.0 mmol/L) or both IFG and IGT. The definitions of IFG and IGT are based on the criteria of the Ministry of Health in Singapore [21]. The study aims to recruit 846 participants across the two arms (423 participants per arm). The outline of the trial design is shown in Fig. 1. This clinical trial follows the guidelines for randomized clinical trials (SPIRIT checklist (Additional file 1)).

### Recruitment

The study participants are referred to the Pre-DICTED program from the following sources:

1. Polyclinics/general practitioner clinics/hospitals
2. Health Promotion Board (HPB) [22]
3. Regional Health System Community Screening programs [23]
4. Self-referral. A call for participants is made on HealthHub, an online portal and mobile application for Singaporeans to access health-related content and their public health records [40].

### Eligibility criteria

Inclusion criteria:

1. Singapore citizens or permanent residents
2. Age between 18 and 64 years
3. BMI ≥ 23.0 kg/m^2^
4. Laboratory tests completed within 3 months of enrolment:
  - FPG 6.1–6.9 mmol/L (IFG) and/or
  - 2-hour plasma glucose in 75 g OGTT 7.8–11.0 mmol/L (IGT)
5. Willing to sign informed consent

Exclusion criteria:

1. Individuals with diabetes mellitus
2. Health conditions impeding participation in lifestyle change program (e.g., active cancer, a recent myocardial event within 6 months, heart failure, chronic kidney disease)
3. Current pregnancy or breastfeeding
4. Intervention with medications known to alter glucose tolerance
5. Known allergic reaction to metformin
6. Active or history of lactic acidosis
7. Severe, acute, or chronic hepatic disease

### Randomization and blinding

Participants are enrolled by study coordinators and randomized according to a 1:1 allocation ratio to the intervention and control arms using an internet-based computer program according to a randomization list. The randomization list was generated using the permuted blocks randomization technique. The block size was determined by a statistician generating the randomization list and will not be disclosed to clinical investigators and other study team members who have contact with study participants. For safety and practical reasons, participants and investigators involved in the stepwise addition of metformin are not blinded after study arm assignment. However, assessment of the primary and secondary outcomes is blinded.

### Study goals

All participants are given 2 study goals: weight loss of ≥ 5% of baseline body weight and moderate-intensity physical activity of ≥ 150 min per week. Both goals have been reported to be safe, feasible, and effective based on previous clinical trials [1, 2].

### Delivery of interventions

Participants in the intervention arm receive structured group-based lifestyle interventions with addition of metformin when needed. They are also eligible for a payment/rewards program. The participants are not allowed to participate in other clinical trials or structured programs involving lifestyle interventions during the study period.

#### Lifestyle interventions

The lifestyle intervention program is based on the Health Promotion Board’s (HPB) pre-diabetes intervention program [25], a public resource portal that includes dietary and physical activity recommendations. The lifestyle intervention program consists of (i) an intensive core intervention phase, delivered through in-person group sessions and worksheet-guided learning, and (ii) a maintenance phase, delivered through mobile phone text messages and telephone calls. Telephone delivery of lifestyle modification intervention has shown the potential to bring about weight loss in obese adults [26] and reduce diabetes incidence in men with impaired glucose tolerance [27] and has the advantage of being less resource-intensive.

##### Core intervention phase (months 1 to 3)

- Weeks 1 to 6: Participants attend 12 group sessions during the first 6 weeks (twice weekly) of the program. This consists of 2 nutrition workshops (2 h per session), 9 exercise sessions (1 h per session), and in the final session a goal-setting workshop (a 2-h session) (Table 1). Group sessions are delivered at community sites in Singapore by a fixed team of facilitators that include dietitians/nutritionists and fitness instructors. The dietitians/nutritionists follow the curriculum provided by HPB and the lessons are repeated for each cohort of participants. For Chinese participants who could not understand English, Mandarin sessions are conducted simultaneously. In the group exercise sessions, fitness instructions gradually increase the exercise intensity over the 9 sessions.
- Weeks 7 to 12: Participants receive worksheets containing information and recommendations on physical activity and nutrition. The worksheets are distributed weekly for 6 weeks of self-directed learning at home. The participants are also given physical activity trackers and digital weighing scales to facilitate self-monitoring of physical activity and weight.

**Table 1:** Pre-DICTED program 12-session core curriculum

**Session 1: Introduction to Pre-DICTED Program and Nutrition Workshop 1: Learn to Eat Healthy today**	
Instil commitment to the Pre-DICTED Program by understanding the importance of lifestyle intervention in diabetes prevention. Highlight the two study goals: 5% weight loss and 150 minutes of weekly physical activity. Understand what diabetes is and how to adopt healthy eating habits as well as how food can affect blood sugars. Introduce tips to manage blood sugar levels through dietary modifications. Learn about food labels and Healthier Choice Symbols. Understand food composition and types of carbohydrates.	
**Session 2: Nutrition Workshop 2: Taking Charge of One’s Meals**	
Explore sugar content in common foods and drinks. Encourage participants to minimize intake of food and drinks with added sugars. Learn about “good fats” and “bad fats”. Learn about the beneficial effects of fiber and sources of fiber. Educate on healthier cooking methods. Introduce tips to self-monitor food intake and build healthy eating habits. Develop a plan for carrying out the changes.	
**Sessions 3 to 11: Exercise sessions: Living a Healthy Life Through Physical Activities**	
Introduce basic principles of physical activity and work towards a minimum of 150 minutes of weekly physical activity over the next 6 weeks. Encourage attendance at group supervised exercise sessions. Experience a range of physical activities, including aerobic and stretching exercises, that is fun and inclusive to invoke continuity in exercising. Learn how to measure heart rate and perceived level of exertion as a way of determining the appropriate levels of activity. Begin self-monitoring of physical activity.	
**Session 12: Goal setting**	
Develop an action plan for physical activity and healthy eating. Learn to find the time to be physically active each day by including short bouts (10–15 min) of activities and record the duration of physical activity. Record food intake and plan for healthy and balanced meals. Start self-monitoring of body weight with intention to achieve study goal of 5% weight loss.	

##### Maintenance phase (months 4 to 36)

During this phase, the participants receive monthly mobile phone text messages on health tips as well as 6-monthly telephone calls from study coordinators to discuss the progress of their lifestyle modifications.

#### Stepwise addition of metformin

The stepwise addition of metformin is done at 6, 12, 18, 24, or 30 months for intervention arm participants who, despite 6 months of lifestyle interventions, are considered to be at the highest risk of conversion to diabetes, defined as having IFG + HbA1c ≥ 6.0% or IFG + IGT. Metformin is prescribed at a starting dose of 250 mg twice daily followed by up-titration to 500 mg twice daily after 3 months if the participants do not experience any side effects. If participants experience side-effects with metformin, the dose is adjusted by the study investigators accordingly. Once added, metformin is prescribed for the remaining study period, ranging from 6 (i.e., added at month 30) to 30 months (i.e., added at month 6). Metformin is discontinued at the end of the study or at the point of diabetes conversion.

#### Financial incentives

In the Pre-DICTED program, participants randomized to the intervention arm are given financial incentives for program adherence and weight loss achievement. Intervention arm participants are given a financial incentive of S$10 (sports vouchers) for attending each group session during the core intervention phase (maximum amount for 12 sessions = S$120). They are also eligible for financial incentives (cash) if they meet the weight loss target (≥ 5% of baseline weight) at any of the study visits at 3, 6, 12, 18, 24, 30 and 36 months. They can receive multiple payments if they maintain the 5% weight loss for subsequent study visits. Payment is interrupted if they fail to maintain the 5% weight loss but they are eligible for payment if they re-meet the target in subsequent visits. The weight loss target is logged-in following the baseline visit and conveyed to the participants. Each participant who meets the weight loss target is given the option of choosing either guaranteed payment or lucky draw payment (1 in 10 chance) according to the schedule in Table 2. The maximum (guaranteed) payment for any participant will be $40 + $50 + $60 + $70 + $80 + $90 + $100 = $490.

**Table 2:** Schedule of guaranteed payment and lucky draw payment for meeting weight loss target (≥ 5% of baseline weight) for intervention arm participants

Month	Guaranteed payments	Lucky draw payments (1 in 10 chance)
3	S$40	S$400
6	S$50	S$500
12	S$60	S$600
18	S$70	S$700
24	S$80	S$800
30	S$90	S$900
36	S$100	S$1,000

### Control group

The current standard of care for pre-diabetes includes referral and follow-up by the participant’s own primary care physician (PCP). The PCPs are instructed to provide individual counseling to the participants on the following topics:

- Education on pre-diabetes
- Importance of lifestyle modifications to prevent progression to diabetes

Participants in the control group follow the same study visits and assessments as those in the intervention group (Table 3). Apart from study visits, participants in the control group will not have additional contact with study coordinators.

**Table 3:** Schedule of study assessments for all participants

Study phase	Screening phase	Follow-up phase							
Visit	Screening								End of study
Scheduled timeline in months (M) (from the date of baseline assessment)	Within -1 M	0 M	3 M	6 M	12 M	18 M	24 M	30 M	36 M
Participant referral	•								
Informed consent	•								
Inclusion/exclusion criteria		•							
Medical history		•							
Demographics		•							
Randomization		•							
***Physical examinations***									
Weight, height, waist circumference		•	•	•	•	•	•	•	•
Heart rate		•	•	•	•	•	•	•	•
Systolic and diastolic blood pressure		•	•	•	•	•	•	•	•
***Laboratory assessments***									
Cholesterol level: Total cholesterol, HDL-c, LDL-c, triglycerides		•		•	•	•	•	•	•
HbA1c		•		•	•	•	•	•	•
Fasting plasma glucose		•		•	•	•	•	•	•
2-hour plasma glucose		•			•		•		•
75 g OGTT		•^a^			•		•		•
***Retinal photography***									
Vision media opacity, diabetic retinopathy, diabetic maculopathy		•			•		•		•
***Questionnaires***									
Food Frequency Questionnaire		•		•	•				
Health status (EQ-5D-5L)		•		•	•	•	•	•	•
Physical activity (IPAQ)		•		•	•	•	•	•	•
Work impairment (WPAI:GH)		•		•	•	•	•	•	•

^a^Participants referred based on FPG alone will need to undergo OGTT at baseline

*HDL-c* high-density lipoprotein-cholesterol, *LDL-c* low-density lipoprotein-cholesterol

### Retention of participants

Appointment reminders have improved attendance in chronic disease follow-up [28], and monetary incentives have demonstrated effectiveness in improving retention in trials [29, 30]. To minimize dropouts, all participants, both controls and those in the intervention group, are given text messaging reminders and telephone call reminders by the study coordinators prior to every study visit. All participants are also asked to make an upfront payment of S$20 as a form of deposit contract. This amount is forfeited if the participant drops out of the project before the end of 3 years or project closeout. The entire amount is quadrupled (i.e., S$80) and returned to the participant if he/she completes the last study visit at the end of the third year. In addition, 6-monthly lucky draws of S$50 sports vouchers (20 vouchers per draw) are conducted for all participants who complete their follow-up visits. They are eligible for multiple draws during the study period.

### Endpoints

#### Primary endpoint

The primary endpoint is the proportion of participants who develop diabetes within 3 years from the baseline visit. Diabetes is diagnosed on the basis of a single FPG of ≥ 7.0 mmol/L or a 2-h plasma glucose of ≥11.1 mmol/L, in accordance with the criteria of the American Diabetes Association [31]. An FPG test is conducted every 6 months, and an OGTT is conducted annually.

#### Secondary endpoints

Secondary endpoints include the change from baseline to end-of-study (3 years) in the following outcome measures:

- HbA1c
- FPG
- Two-hour plasma glucose
- Body weight
- BMI
- Waist circumference
- Dietary intake (assessed by the Food Frequency Questionnaire (FFQ) [32])
- MET-minutes per week (assessed by the International Physical Activity Questionnaire (IPAQ) [33])
- Health status scores (assessed by the EQ-5D-5L [34])
- Work productivity loss and activity impairment (assessed by the Work Productivity and Activity Impairment Questionnaire: General Health V2.0 (WPAI:GH) [35])

Secondary endpoints also include the proportion of participants who have engaged in the targeted level of physical activity (≥ 150 min/week of walking, moderate activity, or vigorous activity as defined by the IPAQ [33]).

### Assessments and visit schedule

Baseline measurements (questionnaire completion, physiological and anthropometric measurement, retinal photography) are taken for all participants. Participants who are referred based on FPG alone also undergo a 75-g OGTT at the baseline visit. Follow-up visits are conducted at month 3 and every 6 months from the date of randomization (6, 12, 18, 24, 30, and 36 months). The acceptable tolerance in the 6-monthly visits is +/− 1.5 months. All assessments are performed by trained study coordinators following standard operational procedures and unaware of participants’ study group allocation. Study visits and assessments are summarized in Table 3. The assessments are described below.

#### Anthropometry

Measurement of body weight, height, waist circumference, and blood pressure is performed at all visits. Height and body weight are measured in light indoor clothing and without shoes, using a portable stadiometer (Model Seca 213, Gmbh & Co.KG, Hamburg, Germany) and a digital weight scale (Model Seca 803, Gmbh & Co.KG, Hamburg, Germany) respectively. Waist circumference is measured at the midpoint between the lower margin of the last palpable rib and the top of the iliac crest, using a stretch-resistant tape. Two waist circumference measurements are made, and the mean value is used. If the two measurements differ by more than 1 cm, both measurements are repeated. Blood pressure and heart rate are measured with the participant in a sitting position after at least 5 min of rest, using a digital blood pressure monitor (Model HEM-7322, Omron Healthcare Co. Ltd, Kyoto, Japan). The first heart rate measurement is used, while the measurement is repeated for blood pressure. If the two measurements differ by more than 5 mmHg for diastolic blood pressure or 10 mmHg for systolic blood pressure, a third measurement is made. The mean value of the closest two blood pressure measurements is used.

#### Physiological measures

For HbA1c, FPG, 2-h plasma glucose in OGTT and lipids profile, participants are instructed to fast overnight for at least 10 h. Blood samples are collected from participants using standard phlebotomy procedures. Plasma glucose is assessed by photometry (ADVIA Chemistry, Siemens, Germany), and HbA1c is assessed by high-performance liquid chromatography (D100 System, Bio-Rad, USA). Serum total cholesterol, HDL-cholesterol and triglycerides are measured by means of enzymatic techniques (ADVIA Chemistry, Siemens, Germany). Additional blood specimens are collected at baseline and month 36. These specimens are stored for future general research beyond the completion of the study.

#### Retinal photography

Retinal photographs are captured using a handheld non-mydriatic fundus camera (Visuscout 100, Zeiss, Germany) by trained technicians. Two fundus images are captured from each eye. The images are subsequently graded for the presence or absence of diabetic retinopathy by trained non-physician graders at a central ocular grading center [36].

#### Questionnaires

##### Dietary intake

Dietary intake is assessed using a locally-validated 163-item semi-quantitative FFQ [32]. The questionnaire consists of a list of 163 food/beverage items, and participants are asked how often they consumed one serving of each item. Data on the responses will be combined with local data on the nutrient content of each FFQ item to calculate the daily intake of major food groups (e.g., vegetables), energy, macronutrients as a percentage of energy intake, and fiber and micronutrient intakes as amount per 1000 kcal.

##### Physical activity

Physical activity is assessed using the short form of the IPAQ [33], a 7-item questionnaire with a recall period of 7 days. It records physical activity at four intensity levels: (i) vigorous activity such as aerobics, (ii) moderate activity such as leisure cycling, (iii) walking, and (iv) sitting. Responses will be used to calculate the total time spent per week on walking, moderate or vigorous activities, and the total MET-minutes per week [33].

##### Health status

Health status is assessed using the EQ-5D-5L [34], a questionnaire consisting of 5 questions with Likert response options (descriptive system) and the EQ visual analog scale (VAS). The EQ-VAS asks participants to rate their own health from 0 to 100 (the worst and best imaginable health, respectively). The descriptive system asks participants to assess the degree of difficulty experienced in the following dimensions: mobility, self-care, usual activities, pain/discomfort, and anxiety/depression. Responses in these 5 dimensions will be used in conjunction with the EQ-5D-3L value set for Singapore [37] to derive the EQ-5D index value. The index value reflects how good a health state is according to the preferences of the general population. A mapping algorithm will be used to link the 5L dimension responses to the 3L value set [38].

##### Work productivity and activity impairment

Work productivity and activity impairment is assessed using the WPAI:GH, a 6-item questionnaire with a recall period of 7 days [35]. The work productivity loss score combines (i) the work time missed due to health problems and (ii) the productivity loss during work time due to health problems (self-reported using a 0 to 10 numerical rating scale). The activity impairment score reflects the degree to which health problems affect regular activities (0 to 10 numerical rating scale).

#### Sociodemographic information

Baseline sociodemographic information is collected to assess baseline imbalance, adjust for potential confounding in the statistical analyses, and stratify participants for subgroup analyses. The information collected include age, ethnicity, education, family history of diabetes, and health history.

### Safety and data monitoring

Internal reviews of adverse events related to metformin are conducted quarterly. In addition, an independent Data Safety and Monitoring Board (DSMB) has been established to review the safety and effectiveness of the study. This Board consists of two clinicians and one statistician, all independent from the trial. The primary objective of the DSMB is to monitor the safety of the interventions and the validity and integrity of the data. The DSMB also evaluates the pace of recruitment and will make recommendations regarding the continuation, modification, or termination of any or all arms of the study.

A planned interim analysis was performed at 2.5 years from the first participant recruitment. Adverse events related to metformin were reported using pre-defined categories: diarrhea, nausea/vomiting, bloatedness, indigestion, weakness, abdominal discomfort, headache, and others. Intervention effectiveness as measured by the primary endpoint (proportion of participants with diabetes) was evaluated using a group sequential method with the O’Brien-Fleming alpha-spending function (overall type I error rate pre-specified at 0.05). An additional interim analysis for safety and effectiveness was performed in February 2021.

### Sample size justification

A study in the Chinese pre-diabetes population showed an 11% annual incidence of diabetes for participants who did not receive any intervention [39], which is equivalent to a 30% incidence of diabetes over 3 years. The study also showed that the relative risk (RR) for lifestyle interventions was 0.64. In addition, the Indian Diabetes Prevention Program study showed that the relative risk (RR) for lifestyle interventions and metformin was 0.72 and 0.74, respectively [5]. Risk ratios for Malays are assumed to fall between those of Indian and Chinese ethnicity. We assumed that the relative risk of converting to diabetes for those receiving intervention in multi-ethnic Singapore (composed primarily of Chinese, Malay, and Indian ethnicities) would be between the estimated effect sizes in the two cited studies; in particular, we used an RR of 0.70 for the intervention.

Given the above assumptions, we aim to recruit up to 846 participants across the two arms (423 participants per intervention arm and control arm). This will allow us to achieve 80% power with 5% two-sided type I error rate and 20% drop-out rate to detect a difference in intervention effect of 10% between the 2 study arms, assuming 33% and 23% incidence of diabetes by 3 years in the control and intervention arm respectively (RR = 0.70). The trial aims to show that the intervention is superior to the current standard of care.

### Data management

Data forms are coded with a study number (starting with P001). Data is entered and stored on a standalone computer and the information is password protected. Only the principal investigator, co-investigators, and study coordinators have access to the data for analysis. Participants are identified by their study number, and only the principal investigator has access to identifiers that link the data to the individual participant. De-identified data is collected and analyzed as specified in the protocol. Participant data will be kept for seven years after the research is completed and all data (electronic and hard copy) will be destroyed after the storage period.

### Statistical analyses

The primary effectiveness analysis will be carried out on the modified intention-to-treat (ITT) population, consisting of all randomized participants who are present for at least one follow-up visit. The risk of prediabetes-to-diabetes conversion at 3 years will be modeled using a generalized linear model (GLM) with an identity link function and binomial variance function. Potential confounders such as age, sex, family history of diabetes and FPG at baseline will be included as covariates. The risk difference between the intervention and control groups and its 95% confidence interval will be reported. The risk of diabetes conversion at months 6, 12, 18, 24, and 30 will also be analyzed using the modified ITT set.

Sensitivity analyses will be performed on the ITT population. Multiple imputation datasets will be generated using chained equations [40], and the imputed datasets will be analyzed using the same GLM and covariates as the primary effectiveness analysis. In addition, two supportive analyses will be conducted: (i) the Kaplan-Meier estimator will be used to compare the cumulative incidence of diabetes in intervention and control participants, and (ii) the proportional hazards model for interval-censored data will be used to analyze the time to diabetes onset. The hazard ratio between the two study arms and its 95% confidence interval will be reported. The same analyses will be performed using the per-protocol population (all randomized participants who (i) have provided FPG and OGTT results at the study conclusion, and (ii) if they are in the intervention arm, have attended the goal-setting workshop, at least one nutrition workshop and at least one exercise session).

Secondary outcomes (engagement in ≥150 minutes of physical activity per week; change from baseline in the following outcomes: HbA1c, FPG, 2-hour plasma glucose, body weight, BMI, waist circumference, health status scores, MET-minutes per week and activity impairment score) will be analyzed using generalized linear models. Subgroup analyses will also be performed, with participants stratified by baseline age (< 50 years, ≥ 50 years), sex, pre-diabetes type (isolated IFG, isolated IGT, IFG + IGT), HbA1c (< 5.7%, 5.7–6.4%, ≥ 6.5%), family history of diabetes, BMI (23.0 to 27.4 kg/m^2^, ≥ 27.5 kg/m^2^), physical activity level (< 150 min/week, ≥ 150 min/week of physical activity), ethnicity (Chinese, Malay, Indian, others), and average monthly income (< S$5000, ≥ S$5000). Baseline characteristics, intervention adherence, and adverse events will be summarized using descriptive statistics.

### Trial management and dissemination policy

The study team includes medical practitioners with expertise in diabetes prevention and treatment, as well as academics with expertise in trial methodology, community-based interventions, health economics, and public health nutrition. The study team discusses recruitment, withdrawal, study progress and adverse events, and will advise on protocol amendments where necessary. Day-to-day running of the trial is managed by the study coordinators. The initial informed consent is taken by the principal investigator, co-investigators, or study coordinators. This includes informed consent for use of biological samples and/or data for future research. Additional informed consent to receive stepwise addition of metformin are taken by the principal investigator or co-investigators who are qualified medical practitioners. The study team will communicate trial results to participants and health professionals via mainstream media and journal publications at the study conclusion.

## Discussion

Lifestyle modification and metformin treatment have been shown in clinical trials to be effective in diabetes prevention and control. However, key challenges remain in the widespread translation and implementation of such programs in real-life community settings with much lower resources. Behavioral economic interventions such as the use of financial incentives may have the potential to improve patient behaviors in such preventive programs. The Pre-DICTED program uses a stepwise model of diabetes prevention consisting of lifestyle interventions and the use of metformin for the highest risk individuals, with added financial incentives to improve attendance at group sessions and to improve weight loss and weight loss maintenance. We aim to test the effectiveness of this treatment strategy in a multi-ethnic population in Singapore. The study will provide data for a future cost-effectiveness analysis, which will be used to inform policymakers in Singapore of the value of a nationwide implementation of the Pre-DICTED program.

## Trial status at time of manuscript submission

The recruitment commenced on 15 Nov 2017 and the recruitment will likely be completed in May 2021. The study uses protocol version 5.0 dated 1 Mar 2019.

## Supplementary Information

**Additional file 1.**

## Data Availability

Not applicable.

## Abbreviations

BMI: Body mass index
DPP: Diabetes prevention program
DSMB: Data Safety and Monitoring Board
FFQ: Food Frequency Questionnaire
FPG: Fasting plasma glucose
GLM: Generalized linear model
HPB: Health Promotion Board
IFG: Impaired fasting glucose
IGT: Impaired glucose tolerance
IPAQ: International Physical Activity Questionnaire
ITT: Intention-to-treat
MET: Metabolic Equivalent of Task
OGTT: Oral glucose tolerance test
RR: Relative risk
WPAI:GH: Work Productivity and Activity Impairment Questionnaire: General Health V2.0
VAS: Visual analog scale
